# “Tocilizumab-an option for patients with COVID-19 associated cytokine release syndrome: A single center experience”, a retrospective study-original article

**DOI:** 10.1016/j.amsu.2021.02.011

**Published:** 2021-02-08

**Authors:** Aijaz Zeeshan Khan Chachar, Khurshid Ahmed Khan, Javeid Iqbal, Adnan Hussain Shahid, Mohsin Asif, Syeda Arzinda Fatima, Asma Afzal Khan, Bilal Bin Younis

**Affiliations:** a Department of Medicine, Fatima Memorial Hospital College of Medicine & Dentistry, Lahore, Pakistan; b Department of Medicine & Endocrinology, Fatima Memorial Hospital College of Medicine & Dentistry, Lahore, Pakistan

**Keywords:** Coronavirus, Covid-19, SARS-CoV-2, Tocilizumab, Cytokine storm syndrome

## Abstract

**Background:**

The first case of Infection with severe acute respiratory syndrome coronavirus 2 (SARS-CoV-2) was diagnosed in Wuhan, China in 2019. In the first half of 2020, this disease has already converted into a global pandemic. This study aimed to find that treatment of patients with COVID-19 pneumonia with Tocilizumab or steroids was associated with better outcomes. **Objectives:** To analyze the effectiveness of Tocilizumab in moderate to severe Covid-19 patients based on predefined assessment criteria***.* Study Settings**: Single-center, Fatima Memorial Hospital, Lahore.

**Study design:**

Quasi-experimental.

**Duration of study:**

From May 12, 2020 to June 12, 2020.

**Patients & Methods: Sample size and technique:**

Sample size was 93; 33 patients were kept in the experimental group, given Tocilizumab, 8 mg/kg intravenously or 162 mg subcutaneously, and the rest of the 60 patients were given corticosteroids, methylprednisolone 80 mg/day. Consecutive sampling. Failure of therapy was labeled when patients were intubated or died, and the endpoints were failure-free survival which was the primary endpoint, and overall survival secondary at the time of discharge.

**Results:**

A total of 93 patients were enrolled, the Tocilizumab (TCZ) group (case) and Corticosteroid (CS) group (Control). The median age was 58 years (IQR-21), 37 (39.8%) patients with diabetes mellitus, 11 (11.8%) in the TCZ group, and 26 (28%) in the CS group. On the whole, the total median hospital stay in days was 7 with IQR (4), a total of 83 (89.2%) patients recovered successfully and discharged, 27 (29%) in the TCZ group and 56 (60.2%) in the CS group. Total 10 (10.8%) patients died, out of which 6 (6.5%) belonged to the TCZ group and 4 (4.3%) belonged to the CS group The median Oxygen requirement with IQR was 8 (9) in both the groups and in total as well, p-value (0.714).

**Conclusions:**

Tocilizumab is a quite effective treatment option for critically sick patients of Covid-19 by reducing their oxygen requirement drastically and so the ICU stay, median hospital stay and so the mortality as well.

**Clinicals trials registration:**

UIN # NCT04730323

## Highlights

• COVID-19 has shaken the world, leaving a huge impact on world’s economy.
• First study in the country assessing effect of Tocilizumab comparing it with corticosteroids.
• Tocilizumab, effectively reduces oxygen requirement, ICU stay, median hospital stay & mortality of COVID-19 sick patients.
• Corticosteroids also emerged as better possible treatment option in the current armamentarium where novel drugs being tried.
• Multiple studies on Tocilizumab conducted worldwide-showing promising results.

## 1 Introduction

The first caseof Infection with severe acute respiratory syndrome coronavirus 2 (SARS-CoV-2) was diagnosed in Wuhan, China in 2019. In the first half of 2020, this disease has already converted into a global pandemic, having different forms of presentation in different patients. An exponential rise in its cases along with associated mortality has shaken the world [1].

Generally, 75% of patients recover without any notable complication however 25% can experience associated complications leading to intensive care unit transfer and even mortality [2]. In some patients of Covid 19, there is an increase in proinflammatory cytokines leading to cytokine storm syndrome. This cytokine storm is mediated by the overproduction of proinflammatory cytokines is seen in a large population of critically ill patients infected with coronavirus disease COVID19 [3]. Patients suffered from cytokine storms present various complications like cardiovascular collapse, multiple organ dysfunctions, and death rapidly [4]. Therefore, early identification, treatment, and prevention of the cytokine storms are of crucial importance for the patients.

There is an urgent need for investigation of the mortality causes and development of novel therapeutic options for severe COVID-19. Anticytokine therapy includes Tocilizumab (TCZ) which has been used worldwide [5].Tocilizumab is one of the first interleukin-6(IL-6) blocking antibodies and has proved its safety and effectiveness in therapy for rheumatoid arthritis. It is a monoclonal antibody and it is being used as an alternative management option for COVID‐19 patients with a risk of cytokine storms [6]. in the present study, we plan to find out the treatment response of TCZ therapy in COVID‐19 infected patients.

COVID-19 is a novel disease-causing infection with the involvement of respiratory symptoms. In the majority of the cases, it is resolved after depicting mild symptoms [7].However severe disease involving multi-organ failure has been seen in a good number of patients. In these patients, a rapid worsening of the clinical condition and biochemical parameters (pro-inflammatory makers) can occur [8].Chest X-rays and computed tomography (CT) scans often demonstrate bilateral large infiltrates. These patients usually need oxygen therapy and sometimes invasive ventilation in the Intensive care unit ICU [9].

Previous studies have demonstrated that in the pathogenesis of severe acute respiratory syndrome (SARS), a cytokine storm occurred, involving a considerable release of proinflammatory cytokine including interleukin-6 (IL-6), tumor necrosis factor-α (TNF-α), and IL-12 [10]. In the research of the Middle East respiratory syndrome (MERS), caused by another coronavirus (MERS-CoV), cytokine genes of IL-6, IL-1β, and IL-8 can be markedly high [11]. A delayed proinflammatory cytokine induction by MERS-CoV was also confirmed. Similar to the changes in SARS and MERS, in COVID-19, higher plasma levels of cytokines including IL-6, IL-2, IL-7, IL-10, granulocyte-colony stimulating factor, interferon-γ (IFN-γ)–inducible protein, monocyte chemoattractant protein, macrophage inflammatory protein 1α, and TNF-α were found in ICU patients, which implied that a cytokine storm occurred and related to the severity and prognosis of the disease [12].

IL-6 receptor (IL-6R) has two forms: membrane-bound interleukin-6 receptor (mIL-6R) and soluble interleukin-6 receptor (SIL-6R) [13]. IL-6 binds to SIL-6R to form a complex, which then binds to gp130 on the cell membrane to complete trans signal transduction and play a proinflammatory role. As a recombinant humanized anti-human IL-6 receptor monoclonal antibody, tocilizumab can specifically bind SIL-6R and mIL-6R and inhibit signal transduction [14]. It is currently used mainly for rheumatoid arthritis [15]. The results of long-term toxicity tests on animals showed that tocilizumab was well tolerated, and no significant abnormalities were observed in other clinicopathological studies or histopathological evaluations [16].

## 2 Objectives

To analyze the effectiveness of Tocilizumab in moderate to severe Covid-19 patients based on predefined assessment criteria.

## 3 Operational definition

### 3.1 Covid-19 infection

Patients were labeled to have Covid-19 infection due to acute respiratory syndrome coronavirus 2 (SARS-CoV-2; previously named as 2019-nCoV) proven via COVID-19 polymerase chain reaction (PCR) test.

## 4 Methodology

### 4.1 Study Design

It was a Quasi-experimental study where participants were allocated using predefined protocol who met the inclusion criteria of study after signing a written & informed consent either by the patient or the person legally authorized to sign.

### 4.2 Study Settings

The study was conducted in Fatima Memorial Hospital, Lahore among in-house patients of COVID-19.

### 4.3 Sample size and technique

Our sample size was 93, out of which 33 patients were kept in the experimental group who were offered Tocilizumab and 60 patients were offered Corticosteroids for comparing the efficacy of both modalities of treatment. The sampling technique was consecutive sampling as per the inclusion and exclusion criteria.

### 4.4 Duration of study

Started after IRB approval, one month. May 12, 2020 to June 12, 2020.

Inclusion Criteria:

• All patients diagnosed with COVID-19 infection with positive reverse transcriptase RT-PCR test, willing to participate in this study or PCR negative patients with clinically COVID-19 Pneumonia in cytokine storm as evidenced by raised inflammatory markers with typical radiological changes
• Patients of both genders were included
• Patients having an age of >65 years with proven Cardiomyopathy, Coronary artery disease, chronic lung disease, Immunosuppressed or organ transplant End-stage renal disease on history & examination and medical records and having any 1 out of 4

Fever 0f.

• ≥39C
• Hypotension or drop in mean arterial pressure of >10 mmHg
• Progressive Hypoxemia requiring >5 L of oxygen
• Sustained Respiratory rate >30/min

With any 2 laboratory parameters out of 3 are present.

D-dimers ≥ 1000 ng/ml.

C-reactive protein CRP ≥100 mg/l

Ferritin ≥600 ng/ml.

• Patients having low risk or no comorbidities and having an age of <65 years with having any 3 out of 4

Fever 0f.

• ≥39C
• Hypotension or drop in mean arterial pressure of >10 mmHg
• Progressive Hypoxemia requiring >5 L of oxygen
• Sustained Respiratory rate >30/min

With any 2 laboratory parameters out of 3 are present.

D-dimers ≥ 1000 ng/ml.

C-reactive protein CRP ≥100 mg/l

Ferritin ≥600 ng/ml.

• Moderate severe or severe COVID 19 features
  1. Shortness of breath oxygen saturation <93% on room air
  2. Progressive Hypoxemia requiring >5 L of oxygen
  3. Respiratory rate >30/min
  4. The partial pressure of arterial oxygen to fraction of inspired oxygen ratio<300
  5. Lung infiltrates on Chest x-ray CXR >50% within 24–48 h
  6. Respiratory failure

Exclusion Criteria:

• Known severe allergic reactions to Tocilizumab or any other monoclonal antibody
• Pregnancy or breastfeeding
• Absolute Neutrophil Count (ANC) < 1000
• Alanine aminotransferase (ALT) or aspartate aminotransferase (AST) > 5 times upper normal limit
• Platelet count of <50,000
• Bowel diverticulitis or bowel perforation
• Patients having Acute pancreatitis

## 5 Data collection and analysis procedure

All admitted patients who had moderate to severe disease or who were in a cytokine storm, were studied and data related to Epidemiological, demographic, clinical, laboratory, radiological, and treatment were collected and analyzed. Outcomes were noted and compared.

The response of the patient after Tocilizumab or Corticosteroid administration was recorded. The response was recorded based on clinical parameters (Oxygen requirement, Fever, Need for invasive positive pressure ventilation), biochemical parameters (D-dimers, C-reactive protein (CRP), Ferritin, Lactate dehydrogenase (LDH) levels), Chest X-ray findings, and Repeated PCR

Test for COVID-19. Any side effects noted after administration of TCZ/Corticosteroid were recorded.

## 6 Tocilizumab administration protocol

Patients received an initial dose calculated as per the body weight (8 mg/kg) maximum 800mg/dose) over 1 h, followed by up to three additional doses if required as per the response after the first dose with 8 h intervals. Predefined Parameters of disease severity were assessed 12 to 24 hourly.(24–72 h). Injection Paracetamol 1g was administered before infusion.

## 7 Corticosteroid administration protocol

Patients received methylprednisolone 80 mg/day in two divided doses as per national/local guidelines and predefined parameters of disease severity were assessed on each day.

Data was entered and analyzed using SPSS 25.0. Frequency and percentages were calculated for the qualitative variables like gender, comorbidity, symptoms, radiological findings, response to treatment. Quantitative variables of the study like age, laboratory parameters were expressed as Median (IQR).

## 8 Ethical considerations

Patients were included in the study after written and informed consent Institutional review board IRB approval was taken from the ethical committee. **IRB# FMH-05**–**2020-IRB-758-M**.

Also registered on clinicals trials. gov # **NCT 04730323**.

## 9 Results

Table 1 summarizes the baseline characteristics of the patients and few important vital signs but few of the important determinants are discussed here. A total of 93 patients were enrolled in our study, two groups were made according to the treatment modality offered and named as Tocilizumab (TCZ) group (case) and Corticosteroid (CS) group (Control), and 33 patients were kept in the TCZ group and 60 patients were enrolled in the corticosteroid group. The median age of all participants was 58 years (IQR-21), TCZ group participants’ median age was 60 years (IQR-14) whereas, in the steroid group, it was 56 years (IQR-23). There were 67 (72%) male participants and 26 (28%) were female. The male predilection of covid-19 was noted down in this study. Regarding age categories, most of the patients 48 (51.6%) were falling in the age range of 50–70 years, followed by 30 (32.3%) and 15 (16.1%) in the age categories of <50 years and >70 years respectively. Regarding the comorbidities in the participants, there were 37 (39.8%) patients with diabetes mellitus, 11 (11.8%) in the TCZ group, and 26 (28%) in the CS group. There were 45 (48.5%) patients having hypertension, 17 (18.3%) in the TCZ group, and 28 (30.1%) in the CS group. There were 9 (9.7%) patients who had chronic kidney disease, 5 (5.4%) and 4 (4.3%) in TCZ and CS group respectively. There were 16 (17.2%) patients who had Ischemic heart disease, 5 (5.4%) and 11 (11.8%) in TCZ and CS group respectively. Discussing lung disease, there were a total of 7 (7.5%) patients and all belonged to the TCZ group only. 48 (51.6%) patients were smokers and 32 (34.4%) in the TCZ group and 16 (17.2%) in the CS group. Patients who had malignancy were 5(5.40%), 2(2.2%), and 3 (3.2%) in the TCZ group and CS group respectively.

**Table 1:** Baseline characteristics of the participants along with Comorbidities, Initial workup and their outcomes.

Characteristics	All patients (N = 93)	Tocilizumab group (N = 33)	*Steroid group (N = 60)*
Median age (IQR)-years	58 (21)	60 (14)	56 (23)
Age category N (%)			
<50 years	30 (32.3)	7 (7.5)	23 (24.7)
50–70 years	48 (51.6)	20 (21.5)	28 (30.1)
>70 years	15 (16.1)	6 (6.5)	9 (9.7)
Male Sex _no (%)	67 (72)	22 (23.7)	45 (48.4)
Female	26 (28)	11 (11.8)	15 (16.1)
*Comorbidities*			
Diabetes Mellitus	37 (39.8)	11 (11.8)	26 (28)
Hypertension	45 (48.5)	17 (18.3)	28 (30.1)
Chronic kidney disease	9 (9.7)	5 (5.4)	4 (4.3)
Ischemic heart disease	16 (17.2)	5 (5.4)	11 (11.8)
Lung disease	7 (7.5)	7 (7.5)	0 (0)
Smoker	48 (51.6)	16 (17.2)	32 (34.4)
Malignancy	5 (5.4)	2 (2.2)	3 (3.2)
*Symptoms at presentation*			
Fever	80 (86.0%)	32 (34.4%)	48 (51.6%)
Cough	75 (80.6%)	29 (31.1%)	46 (49.4%)
Shortness of breath	86 (92.4%)	32 (34.4%)	54 (58.06%)
*Vitals on admission*			
Respiratory rate_ IQR	26 (9)	26 (9)	26 (9)
Heart rate_IQR	110 (19)	109 (11)	112 (10)
Temperature_IQR	100 (99)	101 (99)	100 (99)
*Oxygen saturation at presentation*	90 (13)	90 (13)	90 (12)
*Highest level of Care*			
Intensive care unit	67 (72.04)	33 (35.5)	34 (36.5)
Regular Medical floor	26 (28)	0 (0)	26 (28)
PCR + VE	89 (95.7)	33 (35.5)	56 (60.2) p-value
PCR –VE	4 (4.3)	0 (0)	4 (4.3)
Chest x ray findings			
Bilateral patchy ground glass appearance	22 (23.7)	22 (23.7)	0 (0)
Bilateral Consolidation with peripheral infiltrates	71 (76.3)	11 (11.8)	60 (64.5)
Renal replacement therapy (RRT)	5 (5.4)	1 (1.1)	4 (4.3)
Median hospital stay days (IQR)	7 (4)	5 (3.5)	9 (5)
Outcomes			
Mechanical Ventilation days (IQR)	5 (5.4)	3 (3.2)	2 (2.2)
Death	10 (10.8)	6 (6.5)	4 (4.3)
Recovery	83 (89.2)	27 (29)	56 (60)

IQR, interquartile range; length of hospital stay.

Regarding symptoms on presentation, Shortness of breath was the commonest symptom in both the groups, out of 93 patients, 86 (92.4%) had it, TCZ group and CS group (34.4% vs 58.06%), Fever was observed in 80 (86%), (34.4% vs 51.6%) and cough was seen in 75 (80.6%), (34.4 vs 58.06).

Regarding vitals on admission, the median respiratory rate with IQR was 26 (9) breaths per minute and the same values were seen in both the groups i-e 26 (9) breaths per minute.

The median heart rate was 110 (19) beats per minute, 109 (11) in the TCZ group, and 112 (10) in the CS group.

The median temperature was 100 (2) degrees Fahrenheit.

The Median Oxygen saturation (SPO2%) in liters with the Interquartile range (IQR) at presentation was 90 (13), the same value observed in the TCZ group whereas it was 90 (12) in the CS group.

The highest level of care (Intensive care unit, ICU stay) was offered to a total of 67 (72%) patients according to their severity of the clinical condition, (35.5% vs 34%) in TCZ and CS group respectively.

26 (28%) patients were managed on the medical floor and all these patients belonged to the CS group.

### 9.1 Laboratory & radiologic findings

There were 89 (95.7%) PCR positive patients from their nasopharyngeal swab and only 4 (4.3%) had negative PCR reports but radiologically and clinically they were labeled as PCR negative Covid-19 patients as per decisions of senior consultant radiologists of the hospital and senior physicians.

All patients included in the study underwent a chest radiograph on admission, 22 (23.7%) patients had the bilateral patchy ground-glass appearance and all belonged to the TCZ group and 71 (76.3%) patients had bilateral pulmonary opacities with peripheral infiltrates, 11 (11.8%) vs 60 (64.5%) patients were in TCZ group CS group respectively.

Total 5 (5.4%) patients needed renal replacement therapy (RRT), 1 (1.1%) in TCZ vs 4 (4.3%) in the CS group.

On the whole, the total median hospital stay in days was 7 with IQR (4), there was a shorter hospital stay in the TCZ group, 5 with IQR (3.5) days and a longer hospital stay in CS group 9 with IQR (5) days.

The median time in days of patients who remained on invasive positive pressure ventilation (IPPV), was 5 (5.4) days with a shorter duration in CS group 2 (2.2) and 3 (3.20) in the TCZ group.

Total 83 (89.2%) patients recovered successfully and discharged, 27 (29%) in the TCZ group and 56 (60.2%) in the CS group.

Total 10 (10.8%) patients died, out of which 6 (6.5%) belonged to the TCZ group and 4 (4.3%) belonged to the CS group.

Table .2: showing the comparison between the two groups’ pre and post-treatment.

**Table 2:** Comparative analysis, Pre and Post therapy biochemical markers of Tocilizumab group & Steroids group.

	Variables	Tocilizumab group (N = 33	Steroid group (N = 60)	Total *N93 = )*	p-Value between groups
Before	Oxygen requirement in liters_ Median (IQR)	18 (5)	18 (5)	8 (9)	0.911
	Lymphocytes in 10^9^ cells/liter_ Median (IQR)	1.9 (9.90)	4.6 (4.1)	3.9 (4.0)	0.711
	D-dimers micro gram/mL Median (IQR)	2.1 (4.4)	2.0 (3.60)	2.0 (3.75)	0.844
	CRP mg/dl Median_ (IQR)	200 (190	202 (177)	200 (189)	0.800
	Ferritin ng/ml Median (IQR)	1525 (1255)	2000 (1002)	2000 (1200)	0.424
	Trop I ng/ml _Median (IQR)	16 (17)	12 (11)	13.7 (12.85	0.496
	Serum Creatinine mg/dl_Median (IQR)	1.27 (1.34)	1.31 (1.9)	1.19 (1.1)	0.012
After	Oxygen requirement in liters_ Median (IQR)	8 (9)	8 (9)	8 (9)	0.714
	Lymphocytes in 10^9^ cells/liter_ Median (IQR)	2.9 (2.7)	4 (4.1)	4.8 (4.7)	0.941
	D-dimers micro gram/mL Median (IQR)	1.0 (0.89)	1.0 (1.16)	1.0 (1.16)	0.433
	CRP mg/dl Median_ (IQR)	145 (130)	139 (90)	145 (90)	0.942
	Ferritin ng/ml Median (IQR)	1000 (1266)	1000 (1000)	1000 (1091)	0.884
	Trop I ng/ml _Median (IQR)	16 (18)	11 (11.0)	18 (13)	0.827
	Serum Creatinine mg/dl_Median (IQR)	1.1 (1.2)	1.20 (1.6)	1.4 (1.3)	0.021

Follow up duration 3 days.

Important parameters like Oxygen requirement, Lymphocytes count, biochemical markers like d-dimers, C reactive protein (CRP), serum ferritin levels, Troponin-I, and serum creatinine were measured before and after the treatment was offered to both the group.

### 9.2 Before treatment

The median Oxygen requirement with IQR was 18 (5) in both the groups and in total it was 8 (9). p-value (0.911). The median Lymphocyte count 109 cells/liter 3.9 (4.0), TCZ vs CS group 1.9 (9.9) vs 4.6 (4.1), p-value (0.711), median D-dimers levels microgram/L 2.0(3.75), 2.1(4.4) vs 2.0 (3.60), p-value (0.844), median CRP levels mg/l 200(189), 200(190) vs 202 (177),p-value (0.844), median Ferritin levels ng/ml 2000(1200),1525(1255) vs 2000 (1002),p-value (0.424), median Trop-I levels ng/ml were 13.7(12.85), 16(17) vs 12 (11),p-value (0.496), median Serum creatinine mg/dl was 1.27(1.34), 1.31(1.9) vs 1.19 (1.12), p-value (0.012).

### 9.3 After treatment

The median Oxygen requirement with IQR was 8 (9) in both the groups and in total as well, p-value (0.714). The median Lymphocyte count 109 cells/liter was 4.8 (4.7), TCZ vs CS group 2.9 (2.7) vs 4 (4.1), p-value (0.941), median D-dimers levels microgram/L were 1.0(1.16), 1.0(0.89) vs 1.0 (1.16), p-value (0.433), median CRP levels mg/l was 145(90), 145(130) vs 139 (90),p-value (0.942), median Ferritin levels ng/ml was 1000(1091), 1000(1000) vs 1000 (1091),p-value (0.884), median Trop-I levels ng/ml was 18(13), 16(18) vs 11 (11.0), p-value (0.827), median Serum creatinine mg/dl was 1.4(1.34), 1.1(1.2) vs 1.20 (1.3), p-value (0.021).

## 10 Discussion

In the current study, there was a drop of median 10 L of oxygen requirement in the TCZ group and CS group, which is the significant determinant of the outcome. ICU care was offered to 85% of total patients and almost similar recovery rates were observed in both groups.(40.3% vs 44.8%), and out of 50% of the dead patients, 30% were in the TCZ group and 20% in the CS group, so both treatment modalities were head to head for their successful and almost similar outcomes. In the current study, 80% mortality was seen in the age category of 50–70 years and both the groups shared equal (50%) of expiries.

A landmark study tilted considering cytokine storm and immunosuppression published in early days of COVID-19 pandemic in Lancet 2020 by Mehta P et al. [17] revealed that SARS- Co–V 2 can trigger the immune response enormously, and treating physicians need to be very vigilant to combat this scary cytokine release syndrome, as compared to our study, we had all the mortalities linked with higher levels of inflammatory markers which is the sign of severe cytokine storm syndrome.

A Single-centered retrospective, observational study conducted on 52 critically ill patients in Wuhan, China by Yang X [18] and colleagues concluded that most numbers of ICU admissions were associated with respiratory failure and circulatory shock, as was the result of the retrospective cohort study conducted on 51 patients by Kewan T et al. [19] published in July 2020 that higher levels of inflammatory markers predict the onset of cytokine release syndrome, as compared to our study, our ICU admissions were also 85% due to severity of cytokine release syndrome due to the clinical condition of the patients and higher levels of inflammatory markers in these patients, so our study also comparable with the conclusions of the above-mentioned studies.

A study conducted by Russell CD et al. [20] concluded that corticosteroid treatment was associated with delayed clearance of the virus from the body and recovery may get hampered but in contrast to our study we noticed a good number of patients 56 (60%) recovered from cytokine storm difference can be attributed to the fact that Study by Russell CD and colleagues concluded in the earlier days of Covid-19 pandemic and as the research progressed and we got to know about new trials results favoring the corticosteroid in the resolution of symptoms and an earlier and a better path to recovery, as evidenced by the biggest controlled, open label trial named as RECOVERY trial, promising and the only hope for patients receiving corticosteroids [21].

A study conducted by Kewan T^19^and colleagues measured the interleukin-6 (IL-6)levels to alarm the onset and severity of cytokine release syndrome and as there is no cut off value for IL-6 levels, this study took median IL-6 levels, five times the upper range of normal iL-6 levels for cytokine release syndrome and guide the physicians for appropriate therapy for selected patients but they didn't measure the IL-6 levels serially after infusion of TCZ to monitor the effects and its matter of debate that whether IL-6 levels correlate with the disease severity or not, for the same reason that IL-6 levels weren't performed in this study but there was the definite downtrend of other inflammatory markers like D-dimes and CRP and serum ferritin levels as is the case with our study but the difference in our study was that, we didn't measure interleukin-6 levels in our patients even at the baseline, as it was very costly and not readily available in our hospital settings.

There was sufficient evidence gathered by two major studies, one by XU X [22], et al., published as proceeding of the national academy of science and other Single center retrospective study conducted on fifteen patients by Luo P [23],et al. that there was a drastic reduction in inflammatory markers and settling down of fever after Tocilizumab infusion in patients treated in ICU settings mainly those who were treated with non-invasive ventilation, in contrast to our study our most of the patients (85.1%) from both groups recovered from ICU, Tocilizumab group recovery was 82% and also there was drastic settling down of inflammatory markers performed in our study, which is highly comparable with our study.

A Single center study conducted in Italy on 100 patients by Toniati P et al. [24] revealed that after 24–72 h of Tocilizumab infusion there was a marked reduction in oxygen requirement in patients needing invasive and non-invasive ventilation, as is the case with our study, the median oxygen requirement reduced to 8 L with an IQR of 9 L in both the groups TCZ and CS groups, in same window period (24–72 h). The study conducted by Toniati P [24] and colleagues didn't have any control group so our study was conducted in a better way by keeping the control group as steroid-treated patients to compare the efficacy of the Tocilizumab and corticosteroids.

Another case-control retrospective study conducted by Colaneri M et al. [25] in Italy on 21 Covid-19 patients had the same rate of ICU admissions as those who were given Hydroxychloroquine and azithromycin and in contrast to our study our control group was offered corticosteroid, as a treatment regimen and there were a total of 67 (72.04%) ICU admissions, 33 (35.5%) and 34 (36.5%) in TCZ and CS group respectively, so results are comparable with our study too. However, the study conducted by Colaneri M and colleagues [25] didn't consider the change in oxygen requirements, the major determinant of the outcome, and a recent open letter was published in Lancet [26] stating that there was no use of Hydroxychloroquine with or without macrolide.

In Our study, there was 10 (10.8%) mortality, 6.5% vs 4.3% in TCZ group and CS group, and survival rate was 83 (89.2%), 29% in TCZ group vs 60% in CS group, whereas An observational study conducted in Italy on 85 consecutive patients by Capra R et al. [27] showed a survival benefit in 62 covid-19 patients who were offered a single dose of TCZ and a small number of control 21 patients treated with hydroxychloroquine, lopinavir, and ritonavir which again strengthen and buy our argument of steroids that these are as effective as any modality of treatment.

While discussing the radiological findings in our study, these were described according to the Fleischner Society glossary of terms for thoracic imaging [28]. Increased opacification of lung parenchyma not hiding the blood vessels and bronchi were referred to as ground-glass opacities. Consolidation was labeled on a chest x-ray when there was homogenous opacification of lung parenchyma obscuring blood vessels and bronchi.

Regarding radiological changes, our study had (23.7%) patients who had the ground-glass appearance, and a retrospective descriptive study conducted on 30 patients by Durrani M et, al [29] in Rawalpindi, Pakistan concluded that (23%) patients had ground-glass opacities which is highly comparable with our study, however, our sample size was bigger than the study conducted by Durrani and colleagues.

A multi-center retrospective observational cohort study conducted in 13 hospitals by Biran N et al. [30] included 764 patients needed ICU care and out of those 764 patients, 210 patients received TCZ and this study concluded decreased mortality in patients treated with Tocilizumab as is the case with our study.

## 11 Conclusion

COVID-19 has hit the whole world resulting in significant mortality as well as leaving a huge impact on the world economy. We have found that TCZ is a quite effective treatment option for critically sick patients of Covid-19 by reducing their oxygen requirement drastically and so the ICU stay, median hospital stay and so the mortality as well. Various treatment options are still under study. Corticosteroids also have emerged as a better possible treatment option in the current armamentarium where novel drugs being tried for this novel disease. The role of TCZ is being assessed worldwide as a suitable treatment option for patients with moderate to severe COVID-19. Initial results of multiple studies conducted worldwide have been showing promising results.

## Limitations

It was a single-center study with relatively smaller sample size, but the novelty of the disease outweighed the sample size and it was conducted in one center of Lahore only so results can't be generalized to the whole population of the country unless few other multicenter studies from various provinces of the country reveal similar results as is the case in our study.

## Recommendations

It is highly recommended that further multicenter larger studies need to be conducted after gathering data from all over Pakistan for a better understanding of this novel disease locally as regional variations are quite possible and these implications will not only help the health policy makers to devise a plan but also formulate local guidelines about the treatment regimens and get those implemented it as well to prevent the devastating complications of this novel disease.

## Provenance and peer review

Not commissioned, externally peer-reviewed.

## Disclaimers

None.

## Funding agency

None.

## CRediT authorship contribution statement

**Aijaz Zeeshan Khan Chachar:** Conceptualization, Methodology, Software, Writing - original draft, Writing - review & editing. **Khurshid Ahmed Khan:** Supervision, Project administration. **Javeid Iqbal:** Resources, Data curation. **Adnan Hussain Shahid:** Investigation, Software, Validation. **Mohsin Asif:** Methodology, Formal analysis. **Syeda Arzinda Fatima:** Data curation, Investigation. **Asma Afzal Khan:** Resources, Data curation. **Bilal Bin Younis:** Supervision.

## Declaration of competing interest

Dr. Bilal Bin Younis was the co-author in the study and as he is also the principal of the college so his signatures included in IRB approval letter as his signature is required to issue the IRB approval certificate for everyone applying to get the project approved.
